# Supplementary material for: Cooperative binding of T cell receptor and CD4 to peptide-MHC enhances antigen sensitivity
Source: Nat Commun. 2022 Nov 17;13:7055. doi: 10.1038/s41467-022-34587-w (PMC9671906; doi:10.1038/s41467-022-34587-w)
Supplement: Supplementary file 1 — Supplementary Information [file 41467_2022_34587_MOESM1_ESM.pdf]

Cooperative binding of TCR and CD4 to pMHC enhances TCR sensitivity

Muaz Nik Rushdi[1,2§†], Victor Pan[1,2§‡], Kaitao Li[1,2§], Hyun-Kyu Choi[1,2§], Stefano Travaglino[1,2], Jinsung Hong[1,2¶], Fletcher Griffitts[2,3], Pragati Agnihotri[4,5#], Roy A. Mariuzza[4,5], Yonggang Ke[1,6*], Cheng Zhu[1,2,3*]

[1]Wallace H. Coulter Department of Biomedical Engineering, Georgia Institute of Technology and Emory University, Atlanta, GA, USA; [2]Parker H. Petit Institute for Bioengineering and Biosciences, [3]Georgia W. Woodruff School of Mechanical Engineering, Georgia Institute of Technology, Atlanta, GA, USA; [4]W. M. Keck Laboratory for Structural Biology, Institute for Bioscience and Biotechnology Research, University of Maryland, Rockville, MD, USA; [5]Department of Cell Biology and Molecular Genetics, University of Maryland, College Park, MD, USA; [6]Medical Scientist Training Program, Emory University School of Medicine, Atlanta, GA, USA;

[§]Equally Contributing Authors

[*]Corresponding authors Emails: cheng.zhu@bme.gatech.edu, yonggang.ke@emory.edu

[†]Present Address: Medtronic CO., Minneapolis, MN, USA

[‡]Present Address: Parabon Nanolabs, Huntington, WV, USA

[¶]Present Address: Food and Drug Administration, Silver Spring, MD, USA

[#]Present Address: Advanced Bioscience Laboratories, Rockville, MD, USA

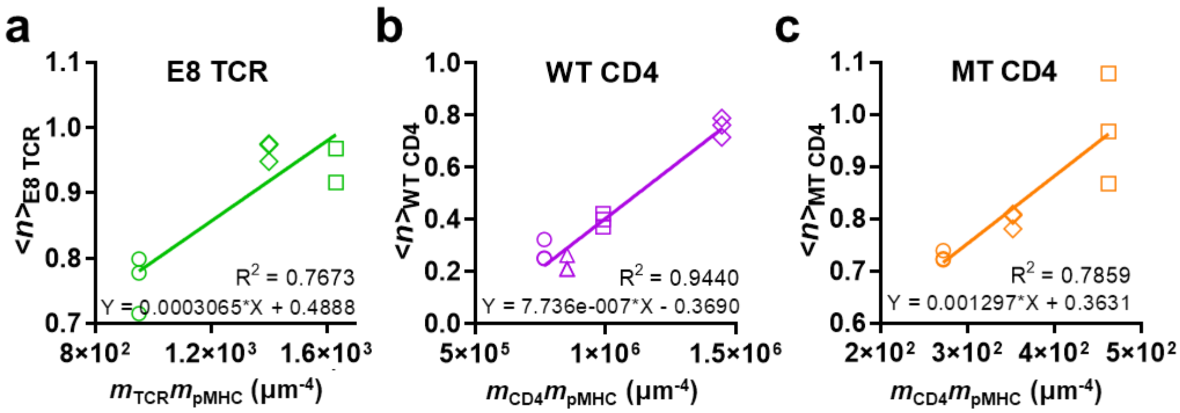

Supplementary Figure 1 | Average number of bonds are proportional to receptor and ligand densities. Related to Fig. 1. Average number of bonds at steady-state vs the product of receptor and ligand densities are fitted by linear regression for pMHC interaction with E8 TCR (**a**), WT CD4 (**b**), or MT CD4 (**c**). The slopes, representing $A_cK_{a,TCR}$, $A_cK_{a,CD4}$, and $A_cK_{a,MT\ CD4}$, respectively, are listed in Table 1.

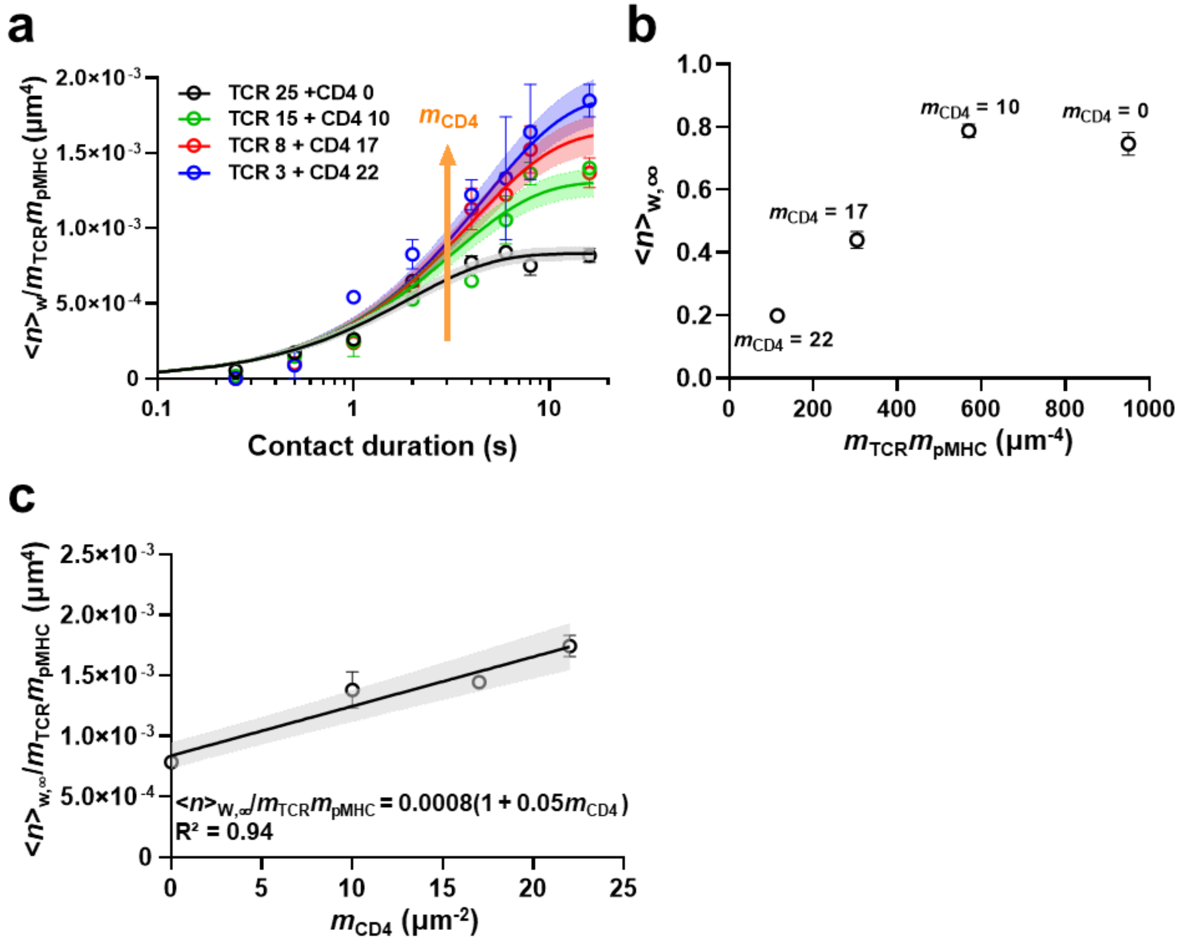

Supplementary Figure 2 | TCR-CD4 cooperativity analysis. Related to Fig. 2b. a, The same data in Figure 2b are plot on semi-log scale. Data points are presented as Mean ± SEM with their respective model fits presented as curves. **b,** Average bond number (Mean ± SEM) at steady-state vs the product of TCR and pMHC densities for data in Figure 2a. **c,** Normalized whole number of bonds (Mean ± SEM) at steady-state is proportional to CD4 density. According to Eq. 3, slope and *y*-intercept represent $K^*_{a,CD4}$ and $A_cK_{a,TCR}$, respectively, and are listed in Table 1.

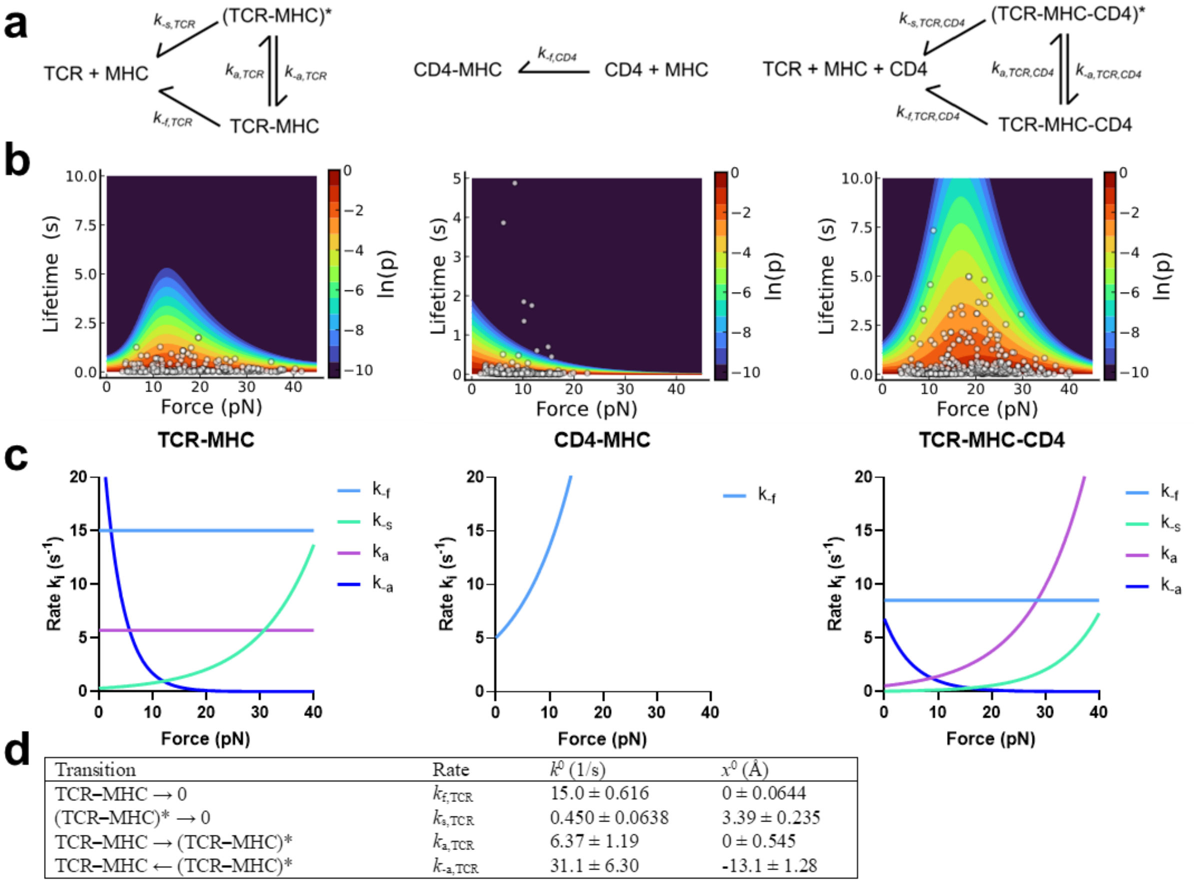

d

Transition	Rate	k^0 (1/s)	x^0 (Å)
TCR–MHC → 0	$k_{f,TCR}$	15.0 ± 0.616	0 ± 0.0644
(TCR–MHC)* → 0	$k_{s,TCR}$	0.450 ± 0.0638	3.39 ± 0.235
TCR–MHC → (TCR–MHC)*	$k_{a,TCR}$	6.37 ± 1.19	0 ± 0.545
TCR–MHC ← (TCR–MHC)*	$k_{-a,TCR}$	31.1 ± 6.30	-13.1 ± 1.28

Transition	Rate	k^0 (1/s)	x^0 (Å)
CD4–MHC → 0	$k_{f,CD4}$	5.03 ± 0.427	4.08 ± 0.387

Transition	Rate	k^0 (1/s)	x^0 (Å)
TCR–MHC–CD4 → 0	$k_{f,TCR,CD4}$	8.53 ± 0.190	0 ± 0.0556
(TCR–MHC–CD4)* → 0	$k_{s,TCR,CD4}$	0.0450 ± 0.00769	5.24 ± 0.223
TCR–MHC–CD4 → (TCR–MHC–CD4)*	$k_{a,TCR,CD4}$	0.544 ± 0.0578	3.98 ± 0.219
TCR–MHC–CD4 ← (TCR–MHC–CD4)*	$k_{-a,TCR,CD4}$	6.80 ± 0.743	-7.83 ± 0.528

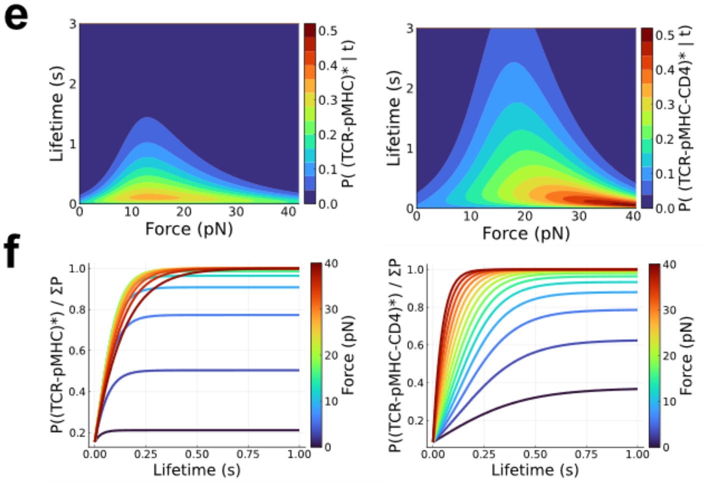

Supplementary Figure 3 | Two-state catch bond models fit the force-dependent lifetime data from experiments. Related to Fig. 2d-f. a, Schematics of two two-state catch bond models and a single-state slip bond model with indicated reaction rates for TCR–pMHC (*left*) and CD4–pMHC (*middle*) bimolecular dissociations, and for the case of TCR–pMHC–CD4 trimolecular dissociation (*right*). The superscript * labels the strong states. **b,** Bond lifetime vs force scattergrams of TCR–pMHC (*left*), CD4–pMHC (*middle*), and TCRαβ + CD4 vs pMHC (*right*) interactions fitted using maximum likelihood estimation (MLE). Depending on the Bell parameters, the three kinetic models generate three families of force-lifetime curves. The likelihoods for data (points) to be observed as predicted (curves) were maximized to obtain the best-fit parameters. Parameters so evaluated from global MLE fitting predict mean ± SE curves of bond lifetime vs force shown in Fig. 2e. They also predict the lifetimes vs force of TCR–pMHC–CD4 trimolecular bonds in the strong state, weak state, and their sum as shown in Fig. 2f. **c,** Reaction rates vs force plots. The force-dependent rates for fast and slow dissociations as well as for activation and deactivation state transitions are plotted for the TCR–pMHC (*left*), CD4–pMHC (*middle*), and TCR–pMHC–CD4 (*right*) bonds. The plots are shown with the best-fit parameters. **d**, Summary of the best-fit Bell parameters used to model the TCR–pMHC (*top*), CD4–pMHC (*middle*), TCR–pMHC–CD4 (*bottom*) dissociations. **e, f**, Probability (e) and fraction (f) of strong bonds as a function of time and force for TCR–pMHC (*left*) and TCR–pMHC–CD4 (*right*) bonds.

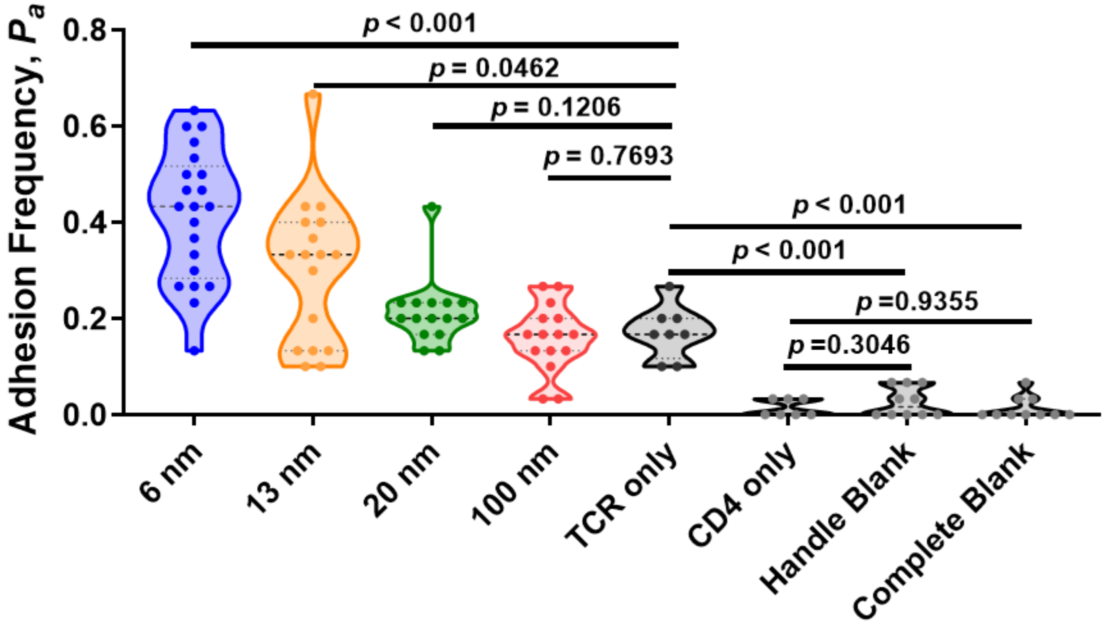

Supplementary Figure 4 | TCR-CD4 cooperativity analysis using DNA origami. Related to Fig. 4. Adhesion frequencies (P_a) of RBCs bearing pMHC interacting with DNA origami beads presenting TCR and CD4 at 6, 13, 20, or 100 nm spacing, or presenting TCR or CD4 alone, or no protein (handle blank and complete blank) at contact time of 4 s (n = 21, 17, 15, 15, 8, 7, 10, and 10 cell-bead pairs). In the violin plots that show data densities, the dashed lines in the middle represent mean and the lower and upper dotted lines represent the first and third quartiles. P-values were calculated for indicated groups using two-tailed Mann-Whitney test.

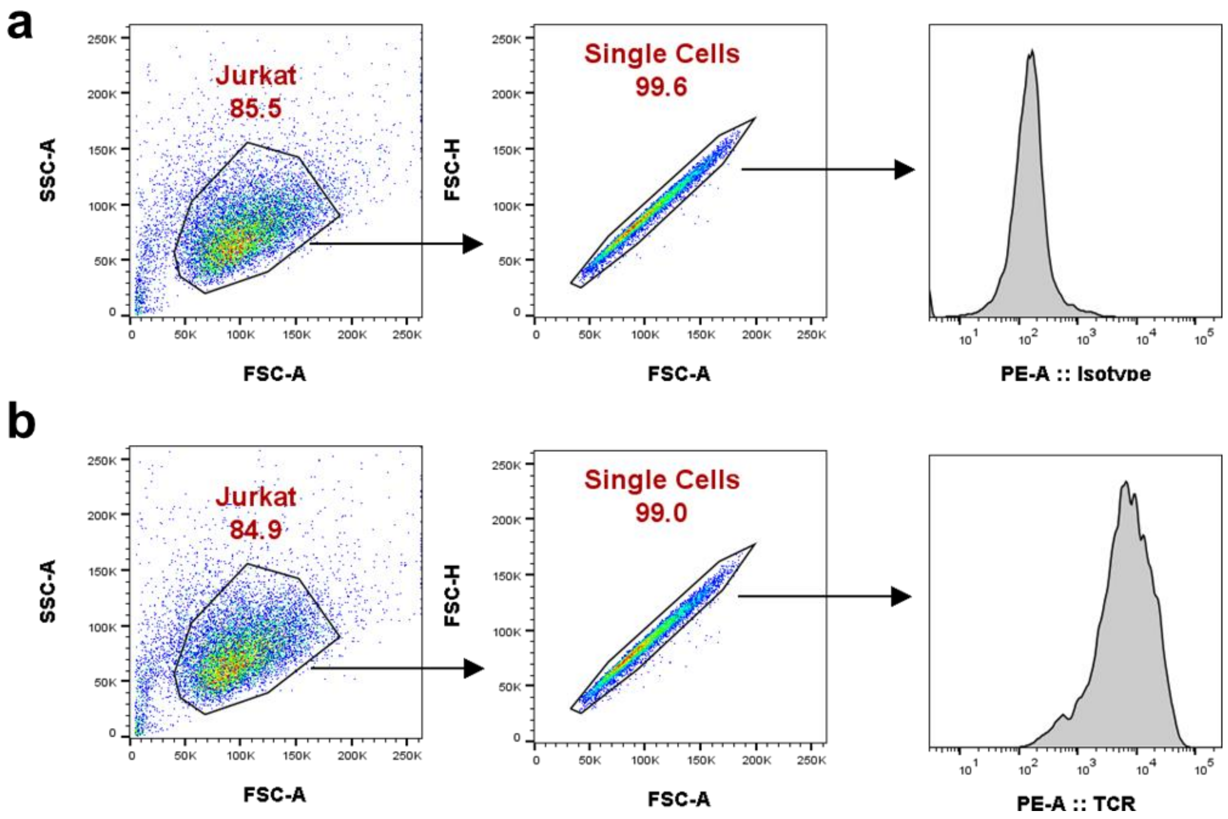

Supplementary Figure 5 | Examples of gating strategy for flow cytometry data analysis. a&b J.RT3 cells expressing E8 TCR and CD4 were stained with PE-anti-human TCR-β1 (**a**) or its isotype control PE-mouse IgG2a κ (**b**). Samples were first gated on FSC-A *vs* SSC-A followed by a secondary gate for single cells based on FSC-A *vs* FSC-H.

Supplementary Table 1 | Site densities of pMHC, TCR, and CD4.

	m_{TCR} (#/μm^2)	m_{CD4} (#/μm^2)	m_{pMHC} (#/μm^2)
Fig. 1b orange	0	16	17
Fig. 1b green	25	n/a	38
Fig. 1b purple	0	900	850
Fig. 1c orange	0	16	17
Fig. 1c green	0	22	16
Fig. 1c purple	0	21	22
Fig. 1d orange square	0	16	22
Fig. 1d orange triangle	0	16	17
Fig. 1d orange circle	0	21	22
Fig. 1d green square	50	0	28
Fig. 1d green triangle	48	0	34
Fig. 1d green circle	25	0	38
Fig. 1d purple square	0	900	1100
Fig. 1d purple triangle	0	900	850
Fig. 1d purple circle	0	1700	850
Fig. 1d purple diamond	0	1550	550
Fig. 2a black	25	0	38
Fig. 2a green	15	10	38
Fig. 2a red	8	17	38
Fig. 2a blue	3	22	38
Fig. 3a green	58	0	19
Fig. 3a purple	0	1948	19
Fig. 3a black	76	96	8
Fig. 3c green	58	0	19
Fig. 3c purple	0	1948	19
Fig. 3c black	76	96	8
Fig. 3d black	14	5	207
Fig. 3d green	25	0	200
Fig. 3d purple	0	10	2832
Fig. 3f green	35	0	n/a
Fig. 3f purple	0	10	n/a
Fig. 3f black	7	7	n/a
Fig. 3f blue	35	9	n/a
Fig. 3h black	14	11	29
Fig. 3h green	42	0	5
Fig. 3h orange	49	53	4
Fig. 3h blue	2	2	52
Fig. 5a blue	35	35	460
Fig. 5a orange	32	32	530
Fig. 5a green	29	29	530
Fig. 5a red	30	30	550
Fig. 5a black-TCR	23	n/a	563
Fig. 5a black-CD4	n/a	23	563
