# Supplementary material for: Cooperative binding of T cell receptor and CD4 to peptide-MHC enhances antigen sensitivity
Source: Nat Commun. 2022 Nov 17;13:7055. doi: 10.1038/s41467-022-34587-w (PMC9671906; doi:10.1038/s41467-022-34587-w)
Supplement: Supplementary file 2 — Reporting Summary [file 41467_2022_34587_MOESM2_ESM.pdf]

nature portfolio

Corresponding author(s): Cheng Zhu

Last updated by author(s): Oct 7, 2022

Reporting Summary

Nature Portfolio wishes to improve the reproducibility of the work that we publish. This form provides structure for consistency and transparency in reporting. For further information on Nature Portfolio policies, see our Editorial Policies and the Editorial Policy Checklist.

Statistics

For all statistical analyses, confirm that the following items are present in the figure legend, table legend, main text, or Methods section.

n/a	Confirmed	
☐	☒	The exact sample size (*n*) for each experimental group/condition, given as a discrete number and unit of measurement
☐	☒	A statement on whether measurements were taken from distinct samples or whether the same sample was measured repeatedly
☐	☒	The statistical test(s) used AND whether they are one- or two-sided<br>*Only common tests should be described solely by name; describe more complex techniques in the Methods section.*
☐	☒	A description of all covariates tested
☐	☒	A description of any assumptions or corrections, such as tests of normality and adjustment for multiple comparisons
☐	☒	A full description of the statistical parameters including central tendency (e.g. means) or other basic estimates (e.g. regression coefficient) AND variation (e.g. standard deviation) or associated estimates of uncertainty (e.g. confidence intervals)
☐	☒	For null hypothesis testing, the test statistic (e.g. *F*, *t*, *r*) with confidence intervals, effect sizes, degrees of freedom and *P* value noted<br>*Give P values as exact values whenever suitable.*
☒	☐	For Bayesian analysis, information on the choice of priors and Markov chain Monte Carlo settings
☒	☐	For hierarchical and complex designs, identification of the appropriate level for tests and full reporting of outcomes
☒	☐	Estimates of effect sizes (e.g. Cohen's *d*, Pearson's *r*), indicating how they were calculated

Our web collection on statistics for biologists contains articles on many of the points above.

Software and code

Policy information about availability of computer code

Data collection	Labview 2016, FACSDiva v9
Data analysis	Labview 2016, Labview 2019, Matlab 2020a, Microsoft Excel 2016, Flowjo v10, GraphPad Prism 8, Julia 1.6, Python 3.7

For manuscripts utilizing custom algorithms or software that are central to the research but not yet described in published literature, software must be made available to editors and reviewers. We strongly encourage code deposition in a community repository (e.g. GitHub). See the Nature Portfolio guidelines for submitting code & software for further information.

Data

Policy information about availability of data

All manuscripts must include a data availability statement. This statement should provide the following information, where applicable:

- Accession codes, unique identifiers, or web links for publicly available datasets
- A description of any restrictions on data availability
- For clinical datasets or third party data, please ensure that the statement adheres to our policy

Data supporting the findings of this study are presented in the article and supplementary materials and are available from the corresponding authors upon reasonable request. Source data are presented for the following figures.
Fig. 1 (b,c,d,e); Fig. 2; Fig. 3; Fig. 4f; Fig. 5; Sup Fig. 1; Sup Fig. 2; Sup Fig. 3 (c,f)

Field-specific reporting

Please select the one below that is the best fit for your research. If you are not sure, read the appropriate sections before making your selection.

☒ Life sciences ☐ Behavioural & social sciences ☐ Ecological, evolutionary & environmental sciences

For a reference copy of the document with all sections, see nature.com/documents/nr-reporting-summary-flat.pdf

Life sciences study design

All studies must disclose on these points even when the disclosure is negative.

Sample size	No sample size calculation was used to predetermine the sample size to be tested. However, expected sample sizes were understood from observing the variance within experiments as well as from leveraging previous publications. For micropipette adhesion frequency assay experiments, repeated 50 contacts generated consistent average adhesion frequencies and therefore between 2 - 9 cell pairs were used as it was understood that adding further cell pairs would not significantly impact the mean values, or the SEM was reported. For BFP experiments, several hundred cell-to-bead or bead-to-bead contacts were tested for each condition. These rules were also adopted in previous publications (refs 9,10,130,15,16, 22,26 and 27).
Data exclusions	For micropipette adhesion frequency assay experiments, no data were excluded from the analysis. For BFP experiments, only clear binding events were used for analysis. in order to extract bond parameters. The profiles of such binding events are detailed in our previous publication (ref. 27).
Replication	Replications were indicated in figure legends. All attempts at replication were successful.
Randomization	For the micropipette adhesion frequency and BFP assays, random pairs were selected from a pool of red blood cells, beads, or cells located in the testing chamber. For all experiments, samples are randomly allocated to each group.
Blinding	The specific DNA origami constructs tested were blinded for the experimenter by the preparer of DNA origami. The identity of the constructs were revealed only upon completion of the micropipette or BFP assays. Samples from other experiments were not blinded due to the complexity of preparation, data collection, and analysis.

Reporting for specific materials, systems and methods

We require information from authors about some types of materials, experimental systems and methods used in many studies. Here, indicate whether each material, system or method listed is relevant to your study. If you are not sure if a list item applies to your research, read the appropriate section before selecting a response.

Materials & experimental systems

n/a	Involved in the study	
☐	☒	Antibodies
☐	☒	Eukaryotic cell lines
☒	☐	Palaeontology and archaeology
☐	☒	Animals and other organisms
☐	☒	Human research participants
☒	☐	Clinical data
☒	☐	Dual use research of concern

Methods

n/a	Involved in the study	
☒	☐	ChIP-seq
☐	☒	Flow cytometry
☒	☐	MRI-based neuroimaging

Antibodies

Antibodies used	The following antibodies used for flow cytometry were bought from BD Biosciences (San Jose, CA): PE anti-human TCR-Cβ1 (clone JOVI.1, 1:20, cat. 566432), PE anti-human CD4 (clone OKT4, 1:20, cat. 566679), PE anti-human HLA-DR (clone L243, 1:5, cat. 340689); Isotype control antibodies were PE mouse IgG2a κ (clone G155-178, 1:20, cat. 555574) for TCR and HLA-DR, and PE mouse IgG2b, κ (Clone 27-35, 1:20, cat. 555741) for CD4. Anti-Mouse CD4 (clone GK1.5, 1:10, cat.553727) was purchased from BD Biosciences.
Validation	JOVI.1, Manufacturer listed reference: Gil D, Schamel WWA, Montoya M, Sanchez-Madrid F, and Alarcon B. Recruitment of Nck by CD3-epsilon reveals a ligand-induced conformational change essential for T cell receptor signaling and synapse formation. Cell. 2002; 109(7):901-912<br>OKT4, Manufacturer listed reference: Courtney AH, Lo WL, Weiss A. TCR Signaling: Mechanisms of Initiation and Propagation. Trends Biochem Sci. 2018; 43(2):108-123. (Biology)<br>L243, Manufacturer listed reference: Edwards JA, Durant BM, Jones DB, Evans PR, Smith JL. Differential expression of HLA class II antigens in fetal human spleen: relationship of HLA-DP, DQ, and DR to immunoglobulin expression. J Immunol. 1986; 137(2):490-7. (Biology)<br>G155-178, Manufacturer listed reference: Prussin C, Metcalfe DD. Detection of intracytoplasmic cytokine using flow cytometry and directly conjugated anti-cytokine antibodies. J Immunol Methods. 1995; 188(1):117-128. (Methodology: Flow cytometry).

27-35, Manufacturer listed reference: Paliard P, Vitrey D, Fournier G, Belhadjali J, Patricot L, Berger F. Perhexiline maleate-induced hepatitis.. Digestion. 1978; 17(5):419-27. (Immunogen).
GK1.5, Manufacturer listed reference: Dialynas DP, Quan ZS, Wall KA, et al. Characterization of the murine T cell surface molecule, designated L3T4, identified by monoclonal antibody GK1.5: similarity of L3T4 to the human Leu-3/T4 molecule. J Immunol. 1983; 131(5):2445-2451. (Immunogen: Blocking, Depletion, Immunoprecipitation).

Eukaryotic cell lines

Policy information about cell lines

Cell line source(s)	HEK293T cells, Jurkat J.RT3 cells, Sf9 cells, and THP-1 cells were from ATCC
Authentication	Authentication was not performed.
Mycoplasma contamination	Mycoplasma contamination was not tested.
Commonly misidentified lines (See ICLAC register)	No commonly misidentified cell lines were used in the study.

Animals and other organisms

Policy information about studies involving animals; ARRIVE guidelines recommended for reporting animal research

Laboratory animals	*For laboratory animals, report species, strain, sex and age OR state that the study did not involve laboratory animals.*
Wild animals	*Provide details on animals observed in or captured in the field; report species, sex and age where possible. Describe how animals were caught and transported and what happened to captive animals after the study (if killed, explain why and describe method; if released, say where and when) OR state that the study did not involve wild animals.*
Field-collected samples	*For laboratory work with field-collected samples, describe all relevant parameters such as housing, maintenance, temperature, photoperiod and end-of-experiment protocol OR state that the study did not involve samples collected from the field.*
Ethics oversight	*Identify the organization(s) that approved or provided guidance on the study protocol, OR state that no ethical approval or guidance was required and explain why not.*

Note that full information on the approval of the study protocol must also be provided in the manuscript.

Human research participants

Policy information about studies involving human research participants

Population characteristics	Healthy male and female (not pregnant) adult donors aged 20 to 40 who weigh at least 110 pounds. Human RBCs were used in this study only as a tool to present biotinylated ligands. The property or biology of RBCs is not the focus of this study.
Recruitment	Participants were recruitment from students and staffs on campus and provided with full disclosure of the usage of the blood. Participant volunteered for blood donation with full consent. There was no self-selection bias.
Ethics oversight	Institutional Review Board of the Georgia Institute of Technology

Note that full information on the approval of the study protocol must also be provided in the manuscript.

Flow Cytometry

Plots

Confirm that:

- ☒ The axis labels state the marker and fluorochrome used (e.g. CD4-FITC).
- ☒ The axis scales are clearly visible. Include numbers along axes only for bottom left plot of group (a 'group' is an analysis of identical markers).
- ☒ All plots are contour plots with outliers or pseudocolor plots.
- ☒ A numerical value for number of cells or percentage (with statistics) is provided.

Methodology

Sample preparation	An aliquot of sample (1 to 5uL depending on cell count) would be added to 100 uL of FACS buffer and antibodies would be added according to dilution factors listed. Sample would be rotated with antibody in the dark for 1 hour. Sample was washed 3X with 500uL of FACS buffer and resuspended in FACS tube with 500uL FACS buffer to be analyzed in flow cytometer.
Instrument	BD Accuri C6 and BD FACSAria

Software	BD Accuri C6 and Flowjo V10
Cell population abundance	A single population of cells were analyzed to quantify the expression level of TCR, CD4, or MHC with single-color staining.
Gating strategy	All cell lines, RBCs, and beads were first gated on FSC/SSC. Gating were based on the expression or coating of molecule of interest.

☒ Tick this box to confirm that a figure exemplifying the gating strategy is provided in the Supplementary Information.
